# Virulence gene profiles: alpha-hemolysin and clonal diversity in *Staphylococcus aureus* isolates from bovine clinical mastitis in China

**DOI:** 10.1186/s12917-018-1374-7

**Published:** 2018-03-02

**Authors:** Limei Zhang, Jian Gao, Herman W. Barkema, Tariq Ali, Gang Liu, Youtian Deng, Sohail Naushad, John P. Kastelic, Bo Han

**Affiliations:** 1 College of Veterinary Medicine, China Agricultural University, Yuan Ming Yuan West Road No. 2, Haidian District, Beijing, 100193 People’s Republic of China; 2 Department of Production Animal Health, Faculty of Veterinary Medicine, University of Calgary, Calgary, AB T2N 4N1 Canada

**Keywords:** *Staphylococcus aureus*, Virulence gene, *Hla*, MLST, *spa*, Bovine mastitis

## Abstract

**Background:**

*Staphylococcus aureus*, a common cause of bovine mastitis, is known for its ability to acquire to antimicrobial resistance and to secrete numerous virulence factors that can exacerbate inflammation. In addition, alpha-hemolysin has an important role in *S. aureus* infections, diversity of the *hla* gene (that produces alpha-hmolysin) in *S. aureus* isolated from bovine mastitis has not been well characterized. The objective was, therefore, to determine diversity of virulence genes, *hla* gene sequences, and clonal profiles of *S. aureus* from bovine mastitis in Chinese dairy herds, and to evaluate inter-relationships.

**Results:**

The antimicrobials resistance varies from as low as 1.9% (2/103) for CTX to as high as 76.7% (79/103) for penicilin in the 103 isolates and 46 (44.7%) *S. aureus* were determined as multi-resistant isolates with diverse resistance patterns. Thirty-eight virulence gene patterns (with variable frequencies) were identified in the 103 isolates and correlated with MLST types, indicating a great diversity. Although the *hla* gene also had great diversity (14 genotypes), Hla peptides were relatively more conserved. With 7 clonal complexes identified from 24 *spa* types and 7 MLST types. Regarding the letter, ST 97 was the dominant type in *S. aureus* from bovine mastitis in China. Furthermore, based on phylogenetic analysis, there was a distinct evolutionary relationship between the *hla* gene and MLST.

**Conclusion:**

Multi-resistant *S. aureus* occurred in bovine mastitis with diverse resistance patterns. The diversity of virulence gene profiles, especially the *hla* gene and, their relationship with molecular types were reported for the first time in *S. aureus* from bovine mastitis, which will be useful for future studies on immunogenicity and vaccine development. In addition, based on the distinct evolutionary relationship between the *hla* gene and MLST types, we inferred that the *hla* gene has potential role for molecular typing of *S. aureus.*

**Electronic supplementary material:**

The online version of this article (10.1186/s12917-018-1374-7) contains supplementary material, which is available to authorized users.

## Background

Bovine mastitis causes huge economic losses in the global dairy industry by decreasing the quality and quantity of milk produced, as well as compromising dairy cow health and welfare. *Staphylococcus aureus*, one of the most prevalent etiologic agents, has an important role in clinical and subclinical mastitis, characterized by persistent and recurrent infections with low cure rates in response to antimicrobial therapy [1–3].

*Staphylococcus aureus* is known for the ability to develop resistance to antimicrobial agents (e.g. methicillin-resistant *S. aureus*, vancomycin-intermediate *S. aureus*, and vancomycin-resistant *S. aureus*) and to secrete numerous virulence factors to exacerbate inflammation. As an alternative to antimicrobials, anti-virulence therapies interfere with bacterial toxins or virulence factors and/or pathways that regulate their production [4]. Allen et al. [5] proposed that a combination of anti-virulence compounds targeting various virulence factors would be a more effective solution than conventional treatments. Therefore, characterization of virulence gene profiles and clonal diversity among *S. aureus* populations are very important in development of anti-virulence therapies [6–9].

Alpha-hemolysin (Hla) toxin is the most emphasized and characterized virulence factor [10] in *S. aureus*. Changes in key amino acid residues may affect Hla activity. For example, a H35L substitution had no hemolytic or lethal activity, whereas a C259T substitution resulted in a premature stop codon and a significant reduction in Hla production [11–13]. Additionally, promising results have been obtained using Hla as a candidate for developing a vaccine to prevent *S. aureus* infections [14–16]. However, variation in Hla peptide sequences could affect vaccine efficacy. It is therefore important to characterize genetic polymorphism of the *hla* gene.

To date, several studies on diversity of *hla* gene of *S. aureus* originating from humans have been reported [13, 17, 18]; however, there is paucity of research regarding sequence variability of this gene in *S. aureus* isolates from bovine mastitis. The objective was to determine virulence gene patterns and *hla* gene diversity in *S. aureus* from bovine mastitis and link them with potential molecular clones, as determined by multilocus sequence type (MLST) and *spa* typing.

## Methods

A total of 103 *S. aureus* isolates from 1021 clinical mastitis samples collected from 2013 to 2016 on 19 dairy farms in 9 provinces of China were used in this study (Table 1). Isolates were identified as *S. aureus* according to conventional methods, including Gram staining, colony morphology, hemolysis, catalase and tube coagulase test, as well as 16S rRNA, *coa* and *nuc* gene sequence analysis, as described [6, 10].

**Table 1:** Distribution of the 103 *Staphylococcus aureus* clinical mastitis isolates according to region and herd

Region	Herd		No. of milk samples	No. of isolates	Total isolates of region
Beijing	a		407	41	48
	b		53	5	
	c		28	2	
Ningxia	d		46	5	16
	e		31	4	
	f		29	3	
	g		28	3	
	h		28	1	
Heilongjiang	i		56	11	11
Hebei	j		38	6	11
	k		23	4	
	l		22	1	
Inner Mongolia	m		25	3	5
	n		22	2	
Liaoning	o		15	2	4
	p		15	2	
Guangdong	q		98	3	3
Shandong	r		40	3	3
Henan	s		17	2	2
Total	19		1021	103	103

### Extraction of genomic DNA

Genomic DNA of all 103 isolates was extracted using Bacteria Genomic DNA Kit (CW, Beijing, China) according to the manufacturer’s instructions and stored at − 20 °C. In addition, quantity and quality of DNA were assessed using a Nanodrop ND-1000 spectrophotometer (Thermoscientific, Wilmington, DE).

### Antimicrobial susceptibility testing

To analyze the antibiotic susceptibility profiles of the 103 isolates, minimum inhibitory concentrations (MICs) of a panel of 11 different antibiotics were determined using the broth microdilution method and resistance breakpoints for ampicillin (AMP, 0.5 mg/L), augmentin (AMX/CA, 32 mg/L), cefotaxime (CTX, 8 mg/L), ceftriaxone (CRO, 8 mg/L), ciprofloxacin (CIP, 4 mg/L), clindamicin (CL, 8 mg/L), erythromycin (E, 8 mg/L), gentamicin (GM, 16 mg/L), oxacillin (OX, 0.5 mg/L), penicillin (P, 0.25 mg/L), tetracycline (TE, 16 mg/L) as described in CLSI (2013) [19]. *S. aurues* ATCC 29213 was used as uality control in this study.

Antimicrobial agents above were selected either according to their availability in commercial products or working as representative of an antibiotic family. And those which were found to be resistant to at least three or more of antimicrobial agents were defined as multidrug resistant isolates.

### Detection of virulence genes

All *S. aureus* isolates were screened by polymerase chain reaction (PCR) for the following virulence genes: fibronectin binding proteins A and B (*fnbA* and *fnbB*), clumping factors A and B (*clfA* and *clfB*), α-, β-, γ-, and δ-hemolysin (*hla*, *hlb, hlc* and *hld*), intracellular adhesion A and D (*icaA* and *icaD*), toxic shock syndrome toxin (*tsst*), and enterotoxins (*sea*, *seb*, *sec*, *see*, *seg* and *sei*). Primer sequences and PCR methods have been described [8, 20, 21]. Products amplified by PCR were sequenced by Beijing Sunbiotech Co. (Beijing, China). Virulence gene profiles were analyzed as binary data using the Maximum Parsimony tree by MEGA6 [22, 23]. Evolutionary distances were computed using the p-distances method and were in units of the number of base differences per site [24].

### Genotyping of *hla* gene and phylogenetic analysis

Primers used for amplification of the complete 960 bp *hla* gene were: *hla*F*1* (5′- TTAGCCGAAAAACATCATTTC -3′) and *hla*R1 (5′- TTATTCCCGACGAAATTCCAA -3′), as described Mocca et al. (2014) [15]. The PCR was performed with initial denaturation at 95 °C for 5 min, followed by 33 cycles of 94 °C for 1 min, 55 °C for 1 min, 72 °C for 1 min, and a final extension at 72 °C for 10 min. Products were sent to Beijing Sunbiotech Co. (Beijing, China) for bidirectional sequencing using both primers: *hla*F2 (5′- GAAGTTATCGGCTAAAGTTATAA -3′) and *hla*R2 (5′- CATAATTAATACCCTTTTTCTC -3′ [15]. All PCR-amplified products were sequenced twice.

*Staphylococcus aureus* strain WOOD46 (GenBank accession no. X01645) was selected as the reference strain, as reported [13, 16, 25–27], and *hla* gene sequences were aligned using BioEdit v7.0.9 (Micro Focus, Newbury, UK) to designate genotypes. Corresponding peptide sequences were deduced and aligned to determine the presence of amino acid substitutions. NetPhosBac 1.0 Server (http://www.cbs.dtu.dk/services/NetPhosBac-1.0) was used to predict serine and threonine phosphorylation sites in Hla peptide sequences, which were identified with non-synonymous amino acid substitutions [28]. In addition, phylogenetic trees of the *hla* gene and 7 MLST alleles in the collection were generated by MEGA6 (http://www.megasoftware.net/) using the neighbor-joining method by the Kimura 2-parameter model with 1000 bootstrap replicates [22].

### Molecular typing

*Staphylococcus aureus* isolates that harbored the *hla* gene were genotyped by staphylococcal protein A (*spa*) typing (http://spa.ridom.de/index.shtml) using SPATypeMapper software (download at http://www.clondiag.com/fileadmin/Media/Downloads/SPATypeMapper_0_6.zip) and multilocus sequence typing (MLST; http://saureus.mlst.net/) [9, 29]. In addition, the goeBURST algorithm (http://goeBURST.phyloviz.net) was used to infer evolutionary associations of MLST types (STs).

### Statistical analyses

Associations between virulence gene profiles, Hla peptide types and clonal background were analyzed using a Chi-square or Fisher’s exact test, as appropriate, on contingency tables (virulence types or Hla peptide types vs clonal backgrounds or geography; virulence types vs Hla peptide types) using SPSS 23.0 (SPSS Inc., Chicago, IL). Statistical significance was defined as *P* < 0.05.

## Results

### Antimicrobial resistance patterns

Overall, there were only 5 *S. aureus* isolates susceptible to all tested antimicrobial compounds, as shown in Table 2. And a total of 46 (44.7%) *S. aureus* were determined as multi-resistant isolates with diverse resistance patterns. In addition, the antimicrobials resistance varies from as low as 1.9% (2/103) for CTX to as high as 76.7% (79/103) for penicillin.

**Table 2:** Antimicrobial resistance patterns of *S. aureus* strains isolated from clinical mastitis in cows

Number of types of resistance	Patterns of resistance	*S. aurues* (*n* = 103)
0 (5)		5
1 (8)	CL	2
	CRO	1
	E	3
	AMP	2
2 (45)	P, AMP	39
	CIP, AMP	1
	OX, E	1
	E, P	1
	CIP, P	1
	OX, AMP	1
	OX, CIP	1
3 (28)	OX, P, AMP	2
	CIP, AMP, P	4
	P, AMP, CL	3
	E, P, AMP	14
	P, AMP, CRO	1
	AMX/CA, P, AMP	1
	OX, E, CL	1
	TE, AMP, P	2
4 (13)	OX, E, CIP, CL	1
	OX, AMX/CA, P, AMP	1
	E, AMX/CA, P, AMP	1
	OX, E, AMP, CL	1
	OX, TE, E, AMP	1
	OX, CIP, P, AMP	1
	E, CIP, AMP, CL	3
	TE, E, P, AMP	1
	OX, E, P, AMP	1
	E, P, AMP, CL	2
≥5 (5)	OX, E, P, CTX, AMP	1
	GM, E, P, AMP, CL	1
	GM, E, CIP, AMP, CL	1
	OX, E, CIP, P, AMP, CL	1
	OX, TE, GM, E, CIP, P, CTX, AMP, CL, CRO	1

*AMP* ampicillin, *AMX/CA* augmentin, *CFX/K* cephalexin/kanamycin, *CIP* ciprofloxacin, *CL* clindamycin, *CRO* ceftriaxone, *CTX* cefotaxime, *E* erythromycin, *GM* gentamicin, *OX* oxacillin, *P* penicillin, *TE* tetracycline

### Virulence gene profiles

**Fig. 1:**
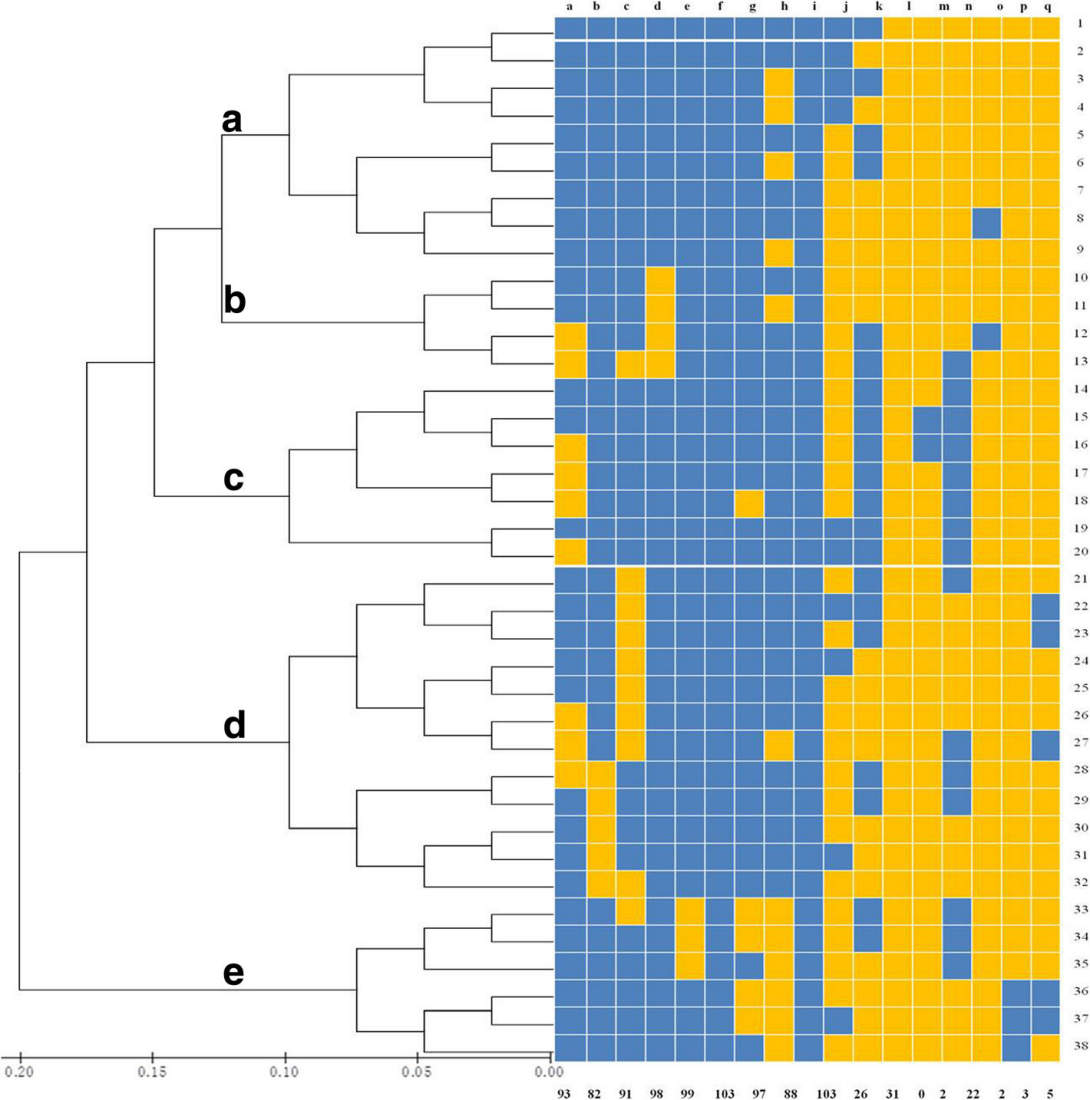
Analysis of virulence gene profiles of 103 Chinese *Staphylococcus aureus* isolates from bovine clinical mastitis. This dendrogram was generated based on the presence/absence of virulence genes with the sum of branch length = 0.91629109. The tree is drawn to scale, with branch lengths in the same units as those of the evolutionary distances used to infer the phylogenetic tree. (**a**-**e**) refer to different virulence gene clusters. Blue boxes indicate the presence and yellow boxes the absence of the corresponding virulence genes, and the number on the right refer to the virulence profile patterns, whereas the number under the box refer to the corresponding number of positive isolates in each virulence gene. (a-q), refers to the virulence genes in order: *hla, hlb, hlc, hld, clfA, clfB, fnbA, fnbB, icaA, icaD, tsst, sea, seb, sec, see, seg, sei*

The PCR amplification results of 17 virulence genes are shown in Additional file 1: Figure S1, Additional file 2: Figure S2, Additional file 3: Figure S3 and Additional file 4: Figure S4, and their relative frequency in 103 clinical mastitis *S. aureus* are shown (Fig. 1). Among them, *clfB* and *icaA* genes were detected in 100% of isolates, followed by *clfA* (*n* = 99; 96%), *hld* (*n* = 98; 95%), *fnbA* (*n* = 97; 94%), *hla* (*n* = 93; 90%), *hlc* (*n* = 91; 88%), *fnbB* (*n* = 88; 85%) and *hlb* (*n* = 82; 80%), *tsst* (*n* = 31; 30%), *icaD* (*n* = 26; 25%), *sec* (*n* = 22; 21%), *sei* (*n* = 5; 5%), *seg* (n = 3; 3%), *seb* and *see* (n = 2; 2%). None of the 103 *S. aureus* isolates had the *sea* gene.

The 103 *S. aureus* isolates comprised 38 virulence gene profiles, which were then categorized into 5 clusters (A to E; Fig. 1). Geographic distribution of these clusters are shown (Table 3). Prevalence of virulence clusters differed among herds from different provinces. Different virulence cluster combinations appeared in herds from different provinces with only 2 exceptions (Herds m and q and Herds s and l; Table 3). Isolates from herds in same province usually had similar cluster combinations. Predominant clusters therefore differed among provinces (*P* < 0.01): Cluster A was the most frequently detected in Beijing herds, Cluster D in herds from Ningxia, and Cluster C in herds from Hebei and Heilongjiang.

**Table 3:** Distribution of virulence gene clusters and Hla peptide sequence types in *Staphylococcus aureus* from 103 cases of clinical mastitis

Province	Herd	Virulence cluster					Hla peptide type									
		A	B	C	D	E	I	II	III	IV	V	VI	VII	VIII	IX	X
Beijing	a	36	2	–	3	–	35	–	3	–	–	–	–	1	1	1
	b	4	–	–	–	1	3	–	–	2	–	–	–	–	–	–
	c	1	1	–	–	–	2	–	–	–	–	–	–	–	–	–
Ningxia	d	–	–	–	5	–	5	–	–	–	–	–	–	–	–	–
	e	–	–	–	4	–	4	–	–	–	–	–	–	–	–	–
	f	–	–	–	3	–	3	–	–	–	–	–	–	–	–	–
	g	–	–	–	3	–	3	–	–	–	–	–	–	–	–	–
	h	–	–	–	1	–	1	–	–	–	–	–	–	–	–	–
Heilongjiang	i	3	–	4	4	–	7	–	–	–	–	1	–	–	–	–
Hebei	j	1	1	1	3	–	4	–	–	–	–	–	–	–	–	–
	k	–	–	4	–	–	2	–	–	–	–	–	–	–	–	–
	l	1	–	–	–	–	1	–	–	–	–	–	–	–	–	–
Inner Mongolia	m	–	–	–	–	3	–	2	–	–	–	–	1	–	–	–
	n	–	–	1	1	–	2	–	–	–	–	–	–	–	–	–
Liaoning	o	1	–	–	1	–	2	–	–	–	–	–	–	–	–	–
	p	1	–	–	1	–	1	–	–	–	–	–	–	–	–	–
Guangdong	q	–	–	–	–	3	1	2	–	–	–	–	–	–	–	–
Shandong	r	1	1	1	–	–	1	–	–	–	–	–	–	–	–	–
Henan	s	2	–	–	–	–	–	–	–	–	2	–	–	–	–	–

- No isolates

### Genotyping based on *hla* gene

Using *S. aureus* strain WOOD 46 as a reference sequence, 22 single nucleotide mutations were detected, 4 in the signal peptide encoding portion (nucleotide positions − 42, − 22, − 6 and − 5) and 18 on the mature *hla* encoding sequence (nucleotide positions 47, 144, 214, 237, 255, 262, 399, 438, 453, 498, 499, 519, 606, 686, 765, 797, 824, 833) (Table 4). As a result 14 *hla* genotypes were identified based on nucleotide sequence analysis and 10 peptide sequence types (I to X) among 93 isolates with the *hla* gene (Tables 4 and 5). Amongst the 14 *hla* genotypes, Genotype 1 contained 65 (70%) isolates (Table 4).

**Table 4:** Nucleotide mutations and corresponding amino acid substitutions of *Staphylococcus aureus* from cases of clinical mastitis

Geno type	No.	Nucleotide mutation position/corresponding amino acid substitution^a^																				
		−42	−22	−6 &-5	47	144	214	237	255	262	399	438	453	498	499	519	606	686	765	797	824	833
		C	G	GG	G	C	G	C	G	T	C	G	T	C	T	T	T	C	T	A	C	T
1	65	−^b^	A	AA	–	–	–	–	–	–	–	–	–	–	–	–	–	–	–	–	–	–
			S^b^	G(−2)N																		
2	5	–	A	AA	–	–	–	–	–	C	T	C	G	–	–	–	C	–	C	–	–	–
			S	G(−2)N						S	S	S	S				S		S			
3	4	T	A	AA	–	–	–	–	–	–	T	C	G	–	–	–	C	–	C	–	–	–
		S	S	G(− 2)N							S	S	S				S		S			
4	4	–	A	AA	–	–	–	–	–	–	–	–	–	T	–	–	–	–	–	–	–	–
			S	G(−2)N										S								
5	3	–	A	AA	–	–	–	–	–	–	–	–	–	–	–	–	–	–	–	–	–	A
			S	G(−2)N																S278Y		
6	2	–	A	AA	–	–	–	–	A	–	–	–	–	–	–	–	–	–	–	–	–	–
			S	G(−2)N					S													
7	2	–	A	AA	–	–	–	–	–	–	–	–	–	–	–	–	–	–	–	–	–	T
			S	G(−2)N																S278F		
8	2	–	A	AA	–	T	–	–	–	–	–	–	–	–	–	–	C	–	C	C	T	–
			S	G(−2)N		S											S		S S T275I			
9	1	–	A	AA	–	–	A	–	A	–	–	–	–	–	–	–	–	–	–	–	–	–
			S	G(−2)N		Q72S			S													
10	1	–	A	AA	T	–	–	–	–	–	T	C	G	–	–	–	C	–	C	–	–	–
			S	G(−2)N	S16I						S	S	S				S		S			
11	1	–	A	AA	–	–	–	A	–	–	–	–	–	–	–	–	–	–	–	–	–	–
			S	G(−2)N				S														
12	1	–	A	AA	–	–	–	–	–	–	–	–	–	–	–	–	–	G	–	–	–	A
			S	G(−2)N													A229G			S278Y		
13	1	–	A	AA	–	–	–	–	–	–	–	–	–	–	–	A	–	–	–	–	–	–
			S	G(−2)N											N173 K							
14	1	–	A	AA	–	–	–	–	–	–	–	–	–	–	D^b^	–	–	–	–	–	–	–
			S	G(−2)N										W167G^c^								

^a^Nucleotide/peptide positions were designated relative to the first nucleotide/amino acid of the mature 훂-hemolysin (*S. aureus* WOOD46)

^b^*S* synonymous mutation, *D* deletion mutation, − = no mutation;

^c^The deletion at nucleotide position 499 of Genotype 14 resulted in peptide termination at residue position 169

**Table 5:** Association between *hla* peptide types and virulence gene profiles of 93 *Staphylococcus aureus* isolated from clinical mastitis

Prediction of phosphorylation sites (n)	Peptide type	*hla*	Virulence gene profile cluster				
			A	B	C	D	E
A (85)	I (77)	1	37	3	6	18	1
		2	0	0	0	5	0
		4	3	0	0	1	0
		6	0	0	1	0	1
		11	1	0	0	0	0
	II (4)	3	4	0	0	0	0
	V (2)	8	2	0	0	0	0
	VII (1)	10	0	0	0	0	1
	IX (1)	13	1	0	0	0	0
B (6)	III (3)	5	2	0	0	1	0
	IV (2)	7	2	0	0	0	0
	VIII (1)	12	1	0	0	0	0
C (1)	VI (1)	9	1	0	0	0	0
	X (1)	14	1	0	0	0	0

Hla peptide type I, composed of *hla* genotypes 1, 2, 4, 6 and 11, was present in 18 herds, peptide sequence type II was in 2 herds, whereas others were identified in only 1 herd with 1 or 2 isolates, indicating that Hla peptide were conserved among herds (Table 3).

**Fig. 2:**
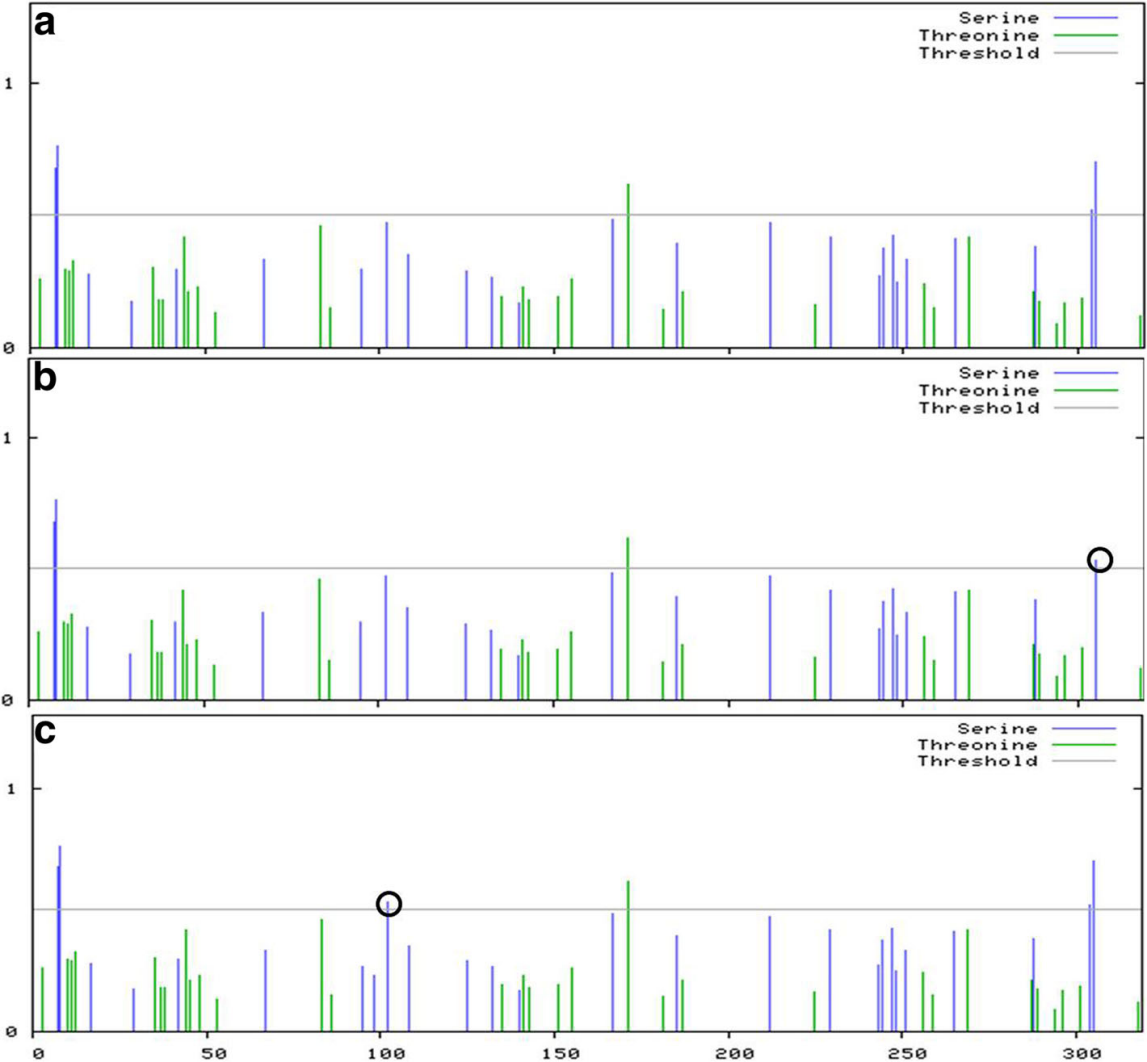
Phosphorylation sites prediction of Hla peptide types with non-synonymous substitutions. The score for each serine or threonine residue is plotted against the sequence position of that residue. When the score is > 0.5, the residue is a predicted phosphorylation site. Variations from the reference sequence are indicated by black circles. **a**) Phosphorylation sites prediction of reference strain WOOD46 and Hla peptide I, II, V, VII, IX; **b**) Phosphorylation sites prediction of Hla peptide type III, IV and VIII; **c**) Phosphorylation sites prediction of Hla peptide type VI and X

Three results were obtained from the prediction of serine and threonine phosphorylation sites among the 92 complete peptide sequences (Fig. 2). Figure 2a presents the prediction results of peptide sequence type I, II, V, VII and IX, which were same as the reference strain. Conversely, there were variations at position 300 in Fig. 2b (results of peptide sequence types III, IV and VIII) and position 100 in Fig. 2c (result of peptide sequence type VI) when comparing with that of the reference strain. Type A was the most dominant in the 93 isolates (*n* = 86, 93%), indicating that the Hla peptide sequence was conserved to an extent (Table 5)

### Molecular typing

The analysis of 103 *S. aureus* isolates by *spa* typing revealed 24 *spa* types, of which 19 were known types (t189, t127, t2699, t359, t237, t4682, t521, t730, t224, t6297, t2756, t131, t1234, t267, t034, t529, t518 and t528), 3 others were reported for the first time (t16314, t16315, and t17095), and 2 were unassigned types with repeats r07r23r12r21r17r34r34r33r34r13 (UK-1) and r07r16r34r33r13r26 (UK-2) (Table 6).

**Table 6:** Distribution of *spa* typing and MLST types of 103 *Staphylococcus aureus* isolated from clinical mastitis in China

Clonal complex (CC)	MLST type	Spa type	^a^BJ			NX					HJ	HB			IM		LN		GD	SD	HN
			a	b	c	d	e	f	g	h	i	j	k	l	m	n	o	p	q	r	s
CC1 (90)	ST188	t189	0	0	0	0	0	0	0	0	0	0	0	0	0	0	0	0	0	0	2
	ST1	t127	0	0	0	0	0	0	0	0	0	0	0	0	0	0	0	0	0	2	0
		t2699	0	0	0	0	0	0	0	0	0	0	0	0	0	0	0	1	0	0	0
		t17095	0	0	0	0	0	0	0	0	0	0	0	0	0	0	1	0	0	0	0
		UK02	0	0	0	0	0	0	0	0	0	0	0	1	0	0	1	0	0	0	0
	ST97	t359	25	1	1	0	0	0	0	0	0	0	0	0	0	0	0	0	0	0	0
		t4570	12	0	0	0	0	0	0	0	0	0	0	0	0	0	0	0	0	0	0
		t237	0	0	0	0	0	0	0	0	6	0	0	0	0	0	0	0	0	0	0
		t4682	0	1	0	0	0	0	0	0	2	0	0	0	0	0	0	0	0	0	0
		t521	0	0	0	0	4	0	0	0	1	0	0	0	0	0	0	0	0	0	0
		t730	0	0	0	0	0	0	0	0	1	3	4	0	0	1	0	0	0	1	0
		t16314	0	2	0	0	0	0	0	0	0	0	0	0	0	0	0	0	0	0	0
		t16315	3	1	0	0	0	0	0	0	0	0	0	0	0	0	0	0	0	0	0
		t224	0	0	0	5	0	0	0	0	0	0	0	0	0	0	0	0	0	0	0
		t6297	0	0	0	0	0	0	3	0	0	0	0	0	0	0	0	0	0	0	0
		t2756	0	0	0	0	0	0	0	1	0	0	0	0	0	0	0	0	0	0	0
		t131	1	0	0	0	0	0	0	0	0	0	0	0	0	0	0	0	0	0	0
		t1234	0	0	1	0	0	0	0	0	0	0	0	0	0	0	0	0	0	0	0
		t267	0	0	0	0	0	0	0	0	1	0	0	0	0	0	0	0	0	0	0
		UK01	0	0	0	0	0	0	0	0	0	1	0	0	0	0	0	0	0	0	0
CC7(1)	ST398	T034	0	0	0	0	0	0	0	0	0	0	0	0	0	0	1	0	0	0	0
CC12(5)	ST705	t529	0	0	0	0	0	0	0	0	0	0	0	0	2	0	0	0	3	0	0
CC22(6)	ST50	t518	0	0	0	0	0	3	0	0	0	2	0	0	0	1	0	0	0	0	0
CC30(1)	ST479	t528	0	0	0	0	0	0	0	0	0	0	0	0	1	0	0	0	0	0	0

^a^*BJ* Beijing, *NX* Ningxia, *HJ* Heilongjiang, *HB* Hebei, *IM* Inner Mongolia, *GD* Guangdong, *SD* Shandong, *HN* Henan, *LN* Liaoning

In addition, 7 MLST types were obtained, which clustered into 7 clonal complexes. Of them, ST97, composed of 82 isolates, was detected in 12 herds from 7 provinces and shared by 15 *spa* types, whereas other MLST types were present in isolates from only 1 or 2 provinces (Table 6). In addition, there was great diversity among isolates within or between herds. Nine of the 19 herds contained > 1 *spa* type (Table 6).

### Phylogenetic tree of *hla* gene

**Fig. 3:**
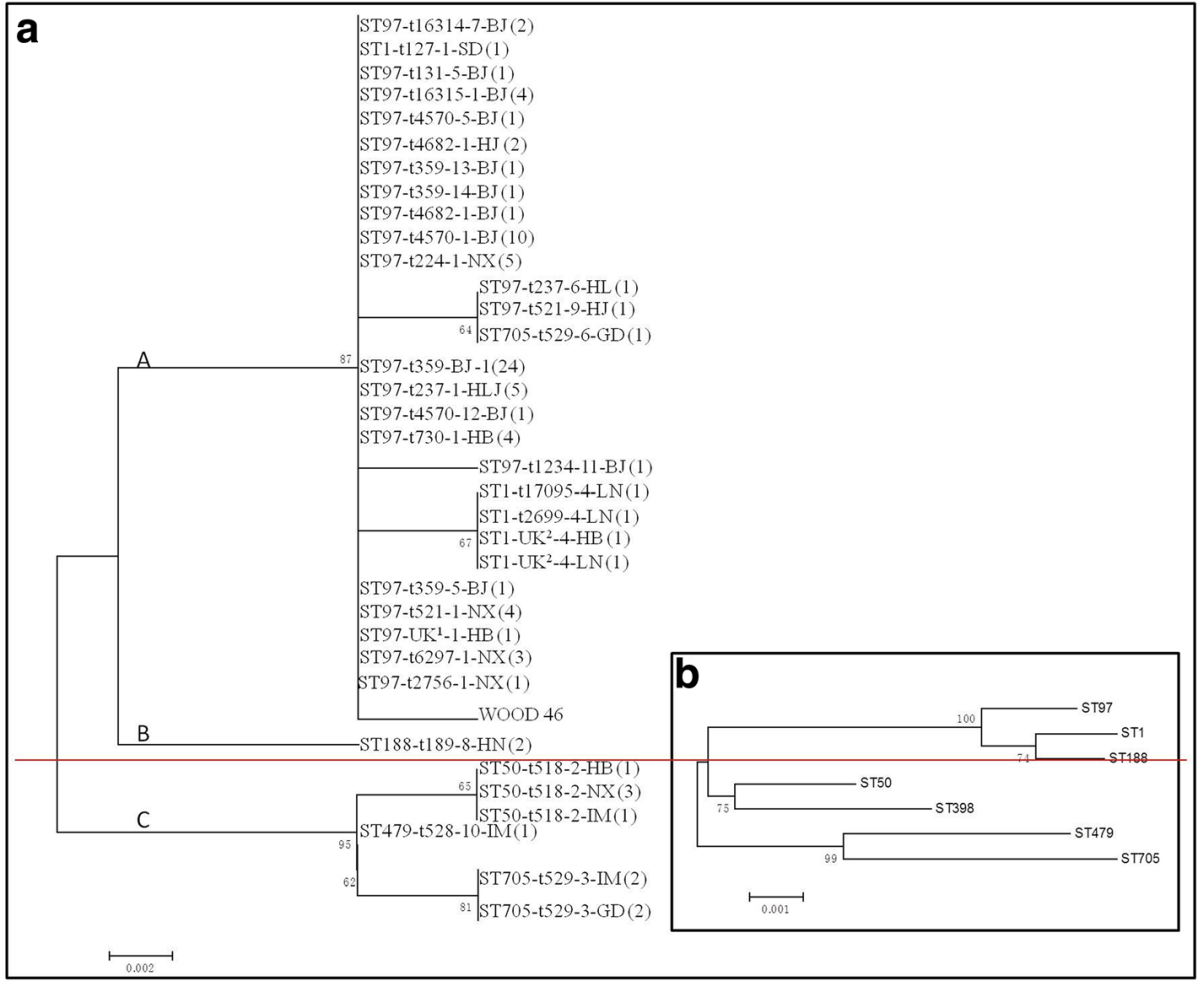
Phylogenetic trees of *hla* gene (**a**) and concatenated sequences of MLST alleles (**b**) in *Staphylococcus aureus* isolates. The percentage of the trees in which the associated *hla* sequence clustered together is shown next to the branches. The tree is drawn to scale, with branch lengths measured in number of substitutions per site. Evolutionary analyses were conducted in MEGA6. Note: BJ = Beijing; NX = Ningxia; SD = Shandong; HB = Hebei; LN = Liaoning; HN = Henan; GD = Guangdong; IM = Inner Mongolia; HJ = Heilongjiang. The number after regions refers to the corresponding *hla* type

According to nucleotide diversity, the 93 *hla* sequences branched into 3 distinct major clusters (Fig. 3a). Furthermore, the corresponding ST types were also grouped into various clusters: ST97 and ST1 were in Cluster A, ST118 was in Cluster B, whereas ST 50, ST705 and ST479 were grouped into Cluster C (Fig. 3a). The position of STs in the tree constructed by the concatenated sequences of the 7 housekeeping genes used in MLST was almost the same as that of *hla* tree, with only ST705 as an exception (Fig. 3b).

### Relationships among virulence gene profiles, *hla* genotypes and clonal typing

Isolates in *hla* type 1/peptide I were present in 5 virulence gene clusters in various proportions, revealing great diversity of virulence genes, whereas isolates in other *hla* genotypes and peptide types demonstrated simplex virulence gene profile (Table 5). Nevertheless, there was no association between *hla* gene genotypes and virulence gene profiles (*P* = 0.12), and no association between Hla peptide types and virulence gene profiles (*P* = 0.80).

Isolates belonging to the same ST may comprise same virulence gene profile. Isolates from ST705 were grouped into Cluster E, whereas ST50 isolates were included in Cluster D. With the exception ST97 and ST1, in which isolates were sprinkled in more than 3 clusters (Table 7). Hence, there was a correlation between virulence gene profiles and molecular clones (*P* < 0.01).

**Table 7:** Association between virulence gene clusters and Hla peptide types, and MLST types and *spa* types

MLST type	*Spa* type	Virulence gene cluster					Hla peptide sequence type									
		A	B	C	D	E	I	II	III	IV	V	VI	VII	VIII	IX	X
ST97	t359, t237, t4682, t521, t730, t16314, t16315, t224, t6297, t2756, t131, t1234, t267, UK-1	45	5	11	20	1	66	0	0	0	1	3	2	1	1	1
ST705	t529	0	0	0	0	5	1	4	0	0	0	0	0	0	0	0
ST50	t518	0	0	0	6	0	5	0	0	0	0	0	0	0	0	0
ST1	t127, t2699, t17095, UK-2	4	0	1	1	0	5	0	0	0	0	0	0	0	0	0
ST479	t528	0	0	0	0	1	0	0	0	1	0	0	0	0	0	0
ST188	t189	2	0	0	0	0	0	0	2	0	0	0	0	0	0	0
ST398	t034	0	0	0	1	0	0	0	0	0	0	0	0	0	0	0

Each Hla peptide sequence type was only present in 1 ST type, except for peptide I, which had isolates in ST97, ST705, ST50, and ST1 (Table 7). Similarly, isolates belonging to the same ST harbored same peptide type, except for ST97, in which isolates were clustered into 7 peptide sequence types (I, V, VI, VII, VIII, IX and X) (Table 5). Therefore, Hla peptide types and ST types were correlated (*P* < 0.01).

## Discussion

It is important to monitor antimicrobial resistance in veterinary medicine. As a frequent and major contagious pathogen in bovine mastitis, *S. aureus* readily becomes resistant to antimicrobials and causes persistent noncurable intramammary infection. Recent investigations on antimicrobial resistance of *S. aureus* are available [30–33]. However, lacking of a common clinical breakpoint for antibiotics which are frequently used to treat mastitis make it difficult to comparing with other data. Overall, the percentage of antimicrobial resistant *S. aureus* in this study is much higher (76.7% vs 25%) in resistant to penicillin according to the work of de Jong et al. [31].

In the present study, the 17 virulence genes were distributed with varying frequencies among *S. aureus* isolates. Consistent with previous research, none of the 103 isolates contained the *pvl* gene [34, 35], whereas adhesin genes (*clfA*, *clfB, fnbA* and *fnbB*) were the most prevalent. In contrast, Gogoi-Tiwari et al. (2015) reported that *fnbB* was only present in 1.3% of *S. aureus* isolates, which was much lower than in our study (85%) [36]. Thompson et al. (2014) reported a lower occurrence of *sec* gene in MSSA than the current study (14 vs 21%, respectively) [37]. In the present study, 30% clinical isolates of *S. aureus* contained the *tsst* gene, whereas in another study [35], none of 47 *S. aureus* mastitis isolates were positive for this gene. In agreement with a previous study, none of the isolates harbored the *sea* gene; this was the gene most frequently encountered from food poisoning in humans and subclinical mastitis cases in cattle [6, 38]. Li et al. (2017) reported that no enterotoxin gene was present in ST97, ST188 or ST398 isolates, which was different from the current study, where some ST97 isolates had *seb*, *sec* and *see* genes [38]. Overall, virulence gene patterns of *S. aureus* isolates had variable distributions among herds from different regions.

There were many reports regarding contribution of α-hemolysin to the pathogenesis of *S. aureus* infection, including cell signaling pathways that govern cell proliferation, cytokine secretion, inflammatory responses and cell-cell interactions [39–42]. Although polymorphisms in the *hla* promoter region have been described [43], the range of genetic diversity and evolution of this toxin was only assessed in human [43–45], with no report of a large representative collection of *S. aureus* from dairy cows. Among the 22 single nucleotide mutations, 14 (64%) mutations were the same as those isolates from humans [13], Among the 22 single nucleotide mutations, 14 (64%) were the same as reported earlier from human isolates, indicating that transmission of *S. aureus* might occur between cows and humans, which would be of great relevance to public health security. The high degree of *hla* gene diversity was in accordance with other reports [17, 23]. However, with the analysis of Hla peptide sequences in this study, we predicted that most (*n* = 86) isolates carried the same phosphorylation site as in our reference strain; therefore, we inferred that Hla peptide sequences were conserved. Although influence of the different phosphorylation sites was uncertain with regards to their influence on the 3-dimensional structure stability of the protein [23], Burnside et al. (2015) reported that a serine/threonine kinase and phosphatase regulated expression of hemolysin in *S. aureus* [46]. Therefore, the exact influence of disparate phosphorylation sites here remains to be studied. Sharma-Kuink et al. (2015) co-related peptide polymorphism to random mutations in nature and selective pressure from the immune system, due to generation of antibodies that bind to alpha-toxins [17]. In this study, the correlation between *hla* gene genotype/ peptides types and geography partly supported the above conclusion. In addition, variations in peptide sequences may influence Hla functionality and may change antigenic epitopes and potentially cause vaccine failure, as reported [15, 16].

Analysis of *hla*-based phylogenetic tree revealed 3 clusters with distinct nucleotide diversity and the ST types of isolates in Cluster A were ST97 and ST1, Cluster B were ST118 and Cluster C were ST 50, ST705 and ST479. Therefore, we inferred that the *hla* gene in *S. aureus* is evolving at varying rates in various genetic backgrounds. This was very similar to a previous study [23]. Interestingly, the tree based on MLST sequences was almost as same as the *hla* tree, revealing that the *hla* gene evolved with MLST background.

Based on analysis of clonal diversity, the 103 *S. aureus* isolates had 24 *spa* types and 7 MLST types, with t359, t730 and ST97 present in most isolates, consistent with previously reports in which ST97 was also the dominant ST type in *S. aureus* from bovine mastitis from China [38, 47]. Conversely, ST239 was reported to be the dominant ST type among 608 hospital-acquired *S. aureus* isolates recovered from human respiratory specimens, sterile body fluids, *S. aureus* bacteremia and non-*S. aureus* bacteremia patients in China [47, 48]. Importantly, 5 new *spa* types (t16314 from Beijing, t16315 from Beijing, t17095 from Liaoning, UK-1 from Hebei and UK-2 from Liaoning and Hebei) were reported for the first time in this study, indicating evolutionary occurrence of unique clones in the region. Most herds harbored at least 3 *spa* types, indicating diversity of the clonal background. When comparing *spa*/ ST types with distribution of isolates, there was a correlation, despite the presence of disparate molecular types.

Notably, there was no correlation between *hla* gene/peptide types and virulence gene clusters. Both virulence gene profile and *hla* gene/peptide types were associated with the molecular clone background, consistent with previous studies in which there was a strong correlation between *hla* gene/ peptide types and clonal background of isolates from human [13]. In addition, there were also differences in virulence genes of various molecular types of strains [38]. Therefore, clone background should be taken into consideration when using Hla as a candidate for vaccines.

The distribution on peptide types, virulence gene profile and molecular clone according to geographical location was not determined, due to the large variation in numbers of *S. aureus* isolates in herds and provinces. However, based on the difference of virulence cluster and *spa* types among herds, we inferred that geography might be an important factor when developing effective treatment strategies for bovine mastitis.

## Conclusions

Multi-resistant *S. aureus* occurred in bovine mastitis with diverse resistance patterns. A great diversity of virulence gene patterns and *spa* typing was determined; *hla* gene evolved with MLST types; ST97 was the dominant types in *S. aureus* from bovine mastitis in China and virulence gene patterns were correlated with MLST types. All of these finding will be useful for future studies on anti-virulence therapies, immunogenicity and vaccine development. In addition, the similarity in diversity of *hla* gene from both humans and bovines make great significance in public health security.

## Additional files

Additional file 1: **Figure S1.** Original picture of PCR result-1: *hla*; 2: *hlb*; 3: *hlc*; 4: *icaD*; 5: *sec*; 6: *sei*; 7: *seg*; 8: *icaA*; 9: *tsst*; 10: *coa*; 11: *nuc*; 12: *clfA*; 13: *clfB*; 14: *fnbA*; 15: *fnbB. (ZIP 31 kb)*

Additional file 2: **Figure S2.** Original picture of PCR result of *hld* gene. (JPEG 39 kb)

Additional file 3: **Figure S3.** Original picture of PCR result of *seb* gene. (JPEG 7 kb)

Additional file 4: **Figure S4.** Original picture of PCR result of *see* gene. (JPEG 5 kb)

## Abbreviations

AMP: Ampicillin
AMX/CA: augmentin
CIP: Ciprofloxacin
CL: Clindamicin
*clfA*: clumping factors A
*clfB*: clumping factors B
CRO: Ceftriaxone
CTX: Cefotaxime
E: Erythromycin
*fnbA*: fibronectin binding proteins A
*fnbB*: fibronectin binding proteins B
GM: Gentamicin
Hla: Alpha-hemolysin
*hla*: α- hemolysin
*hlb*: β-hemolysin
*hlc*: γ-hemolysin
*hld*: δ-hemolysin
*icaA*: intracellular adhesion A
*icaD*: intracellular adhesion D
MICs: Minimum inhibitory concentrations
MLST: Multilocus sequence type
OX: Oxacillin
P: penicillin
PCR: Polymerase chain reaction
*S. aureus*: *Staphylococcus aureus*
*sea*: *Staphylococcus aureus* enterotoxin a
*sec*: *Staphylococcus aureus* enterotoxin c
*see*: *Staphylococcus aureus* enterotoxin e
*seg*: *Staphylococcus aureus* enterotoxin g
*sei*: *Staphylococcus aureus* enterotoxin i
*spa*: staphylococcal protein A
STs: MLST types
TE: tetracycline
*tsst*: toxic shock syndrome toxin
