# Supplementary material for: Virulence gene profiles: alpha-hemolysin and clonal diversity in Staphylococcus aureus isolates from bovine clinical mastitis in China
Source: BMC Vet Res. 2018 Mar 2;14:63. doi: 10.1186/s12917-018-1374-7 (PMC5834907; doi:10.1186/s12917-018-1374-7)
Supplement: Supplementary file 1 — Figure S1. Original picture of PCR result-1: hla; 2: hlb; 3: hlc; 4: icaD; 5: sec; 6: sei; 7: seg; 8: icaA; 9: tsst; 10: coa; 11: nuc; 12: clfA; 13: clfB; 14: fnbA; 15: fnbB. (ZIP 31 kb) [file 12917_2018_1374_MOESM1_ESM.zip › Figure S1-Original picture of PCR results of 15 genes note.docx]

**Supplement Figure1.** PCR picture of 15 virulence genes

1: *hla*; 2: *hlb*; 3: *hlc*; 4: *icaD*; 5: *sec*; 6: *sei*; 7: *seg*; 8: *icaA*; 9: *tsst*; 10: *coa*; 11: *nuc*; 12: *clfA*; 13: *clfB*; 14: *fnbA*; 15: *fnbB*.
