# Supplementary figures and images for: Virulence gene profiles: alpha-hemolysin and clonal diversity in Staphylococcus aureus isolates from bovine clinical mastitis in China
Source: BMC Vet Res. 2018 Mar 2;14:63. doi: 10.1186/s12917-018-1374-7 (PMC5834907; doi:10.1186/s12917-018-1374-7)

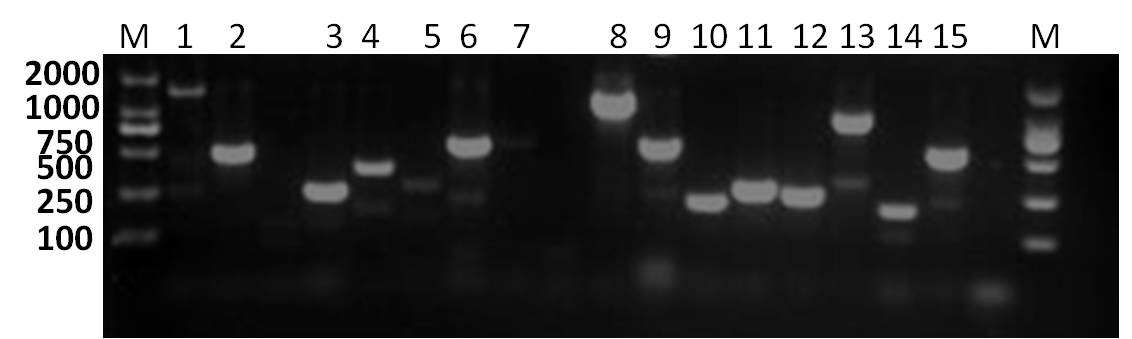

Supplement: Supplementary file 1 — Figure S1. Original picture of PCR result-1: hla; 2: hlb; 3: hlc; 4: icaD; 5: sec; 6: sei; 7: seg; 8: icaA; 9: tsst; 10: coa; 11: nuc; 12: clfA; 13: clfB; 14: fnbA; 15: fnbB. (ZIP 31 kb) [file 12917_2018_1374_MOESM1_ESM.zip › Figure S1-Original picture of PCR results of 15 genes.jpg]

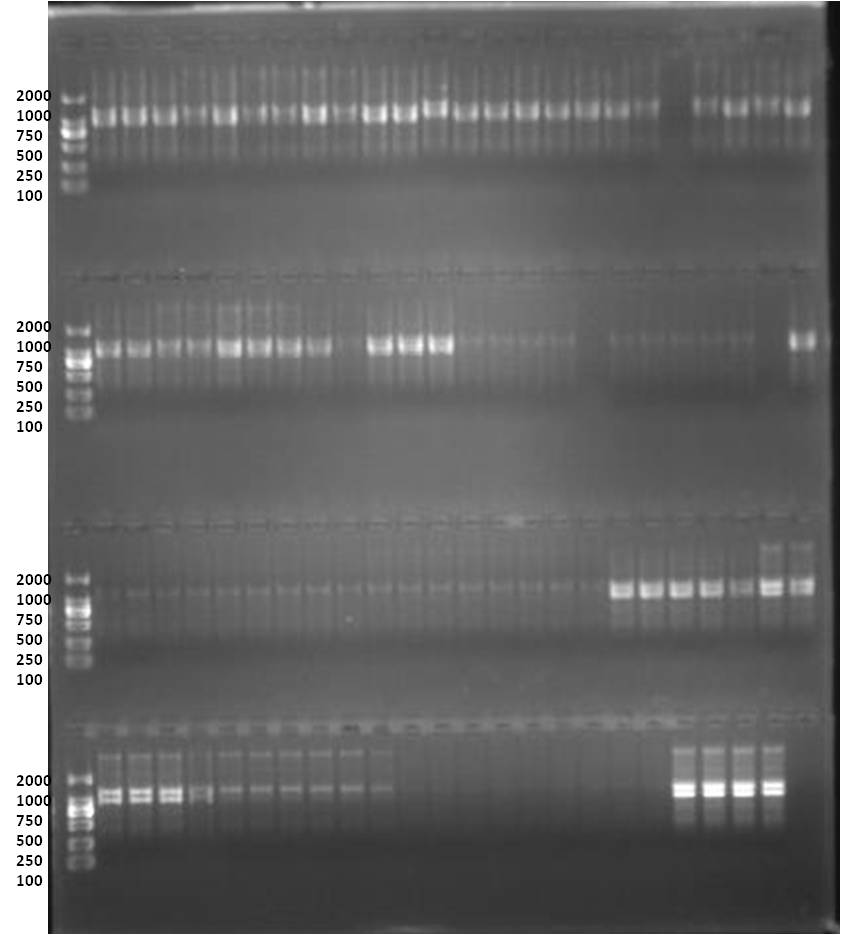

Supplement: Supplementary file 2 — Figure S2. Original picture of PCR result of hld gene. (JPEG 39 kb) [file 12917_2018_1374_MOESM2_ESM.jpg]

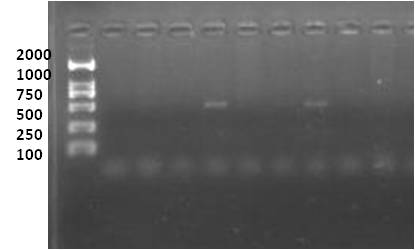

Supplement: Supplementary file 3 — Figure S3. Original picture of PCR result of seb gene. (JPEG 7 kb) [file 12917_2018_1374_MOESM3_ESM.jpg]

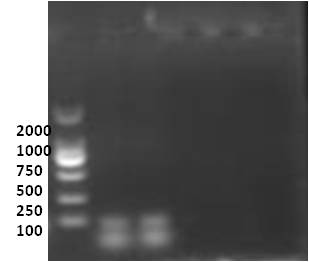

Supplement: Supplementary file 4 — Figure S4. Original picture of PCR result of see gene. (JPEG 5 kb) [file 12917_2018_1374_MOESM4_ESM.jpg]
